# Correction: Incidence of Lyme disease in the United Kingdom and association with fatigue: A population-based, historical cohort study

**DOI:** 10.1371/journal.pone.0274408

**Published:** 2022-09-06

**Authors:** Florence Brellier, Mar Pujades-Rodriguez, Emma Powell, Kathleen Mudie, Eliana Mattos Lacerda, Luis Nacul, Kevin Wing

## Notice of Republication

In light of an issue identified post-publication, this article was republished on May 11, 2022. Please download this article again to view the correct version.
