# Supplementary material for: Incidence of Lyme disease in the United Kingdom and association with fatigue: A population-based, historical cohort study
Source: PLoS One. 2022 Mar 23;17(3):e0265765. doi: 10.1371/journal.pone.0265765 (PMC8942220; doi:10.1371/journal.pone.0265765)
Supplement: S3 Table — (DOCX) [file pone.0265765.s003.docx]

S3 Table - Incidence rates, crude and adjusted hazard ratios (HR) of any types of fatigue and ME/CFS for Lyme disease using an extended cohort including also suspected Lyme disease patients and their matched controls.

|  | Participants with outcomes | Total  person-years | Incidence rates^b^ | Crude HR^c^ (95% CI) | Adjusted HR^d^ (95% CI) | p value^d^ |
| --- | --- | --- | --- | --- | --- | --- |
| Any types of fatigue^a^ | | | | | | |
| Comparator cohort (non-Lyme) (N=10,274) | 720 | 42,828 | 168.12 |  |  |  |
| Lyme cohort (N=2,571) | 317 | 10,061 | 315.08 | 2.29 (1.99-2.65) |  |  |
| January-February-March |  |  |  |  | 2.22 (1.24-3.97) | 0.007 |
| April-May-June |  |  |  |  | 1.69 (1.08-2.65) | 0.022 |
| July-August-September |  |  |  |  | 1.59 (1.11-2.29) | 0.012 |
| October-November-December |  |  |  |  | 3.20 (1.98-5.20) | <0.001 |
| Chronic Fatigue Syndrome | | | | | | |
| Comparator cohort (non-Lyme) (N=10,274) | 5 | 45,347 | 1.10 |  |  |  |
| Lyme cohort (N=2,571) | 13 | 11,168 | 11.64 | 10.57 (3.77-29.66) | 20.18 (6.35-64.15) | <0.001 |
| ^a^symptoms of fatigue, post-viral fatigue, or chronic fatigue syndrome; ^b^per 10,000 person-years; ^c^using Cox regression with adjustment for the match variable (i.e. age, sex, and general practice) for any types of fatigue and no adjustement for ME/CFS; ^d^robust standard errors, adjusting additionally for healthcare utilisation frequency as a continuous variable, prescribed antibiotic treatment, and season index as effect modifier for any types of fatigue and for prescribed antibiotic treatment for Chronic Fatigue Syndrome; | | | | | | |
