# Supplementary material for: Incidence of Lyme disease in the United Kingdom and association with fatigue: A population-based, historical cohort study
Source: PLoS One. 2022 Mar 23;17(3):e0265765. doi: 10.1371/journal.pone.0265765 (PMC8942220; doi:10.1371/journal.pone.0265765)
Supplement: S4 Table — (DOCX) [file pone.0265765.s004.docx]

S4 Table - Incidence of any types of fatigue and ME/CFS in patients with multiple infections with bacterium *Borrelia burgdorferi*

|  | Total number of patients^a^ | Patients with outcomes  "any types of fatigue^b^” | Percentage of patients with any types of fatigue | Patients with outcomes  "Chronic Fatigue Syndrome" | Percentage of patients with Chronic Fatigue Syndrome |
| --- | --- | --- | --- | --- | --- |
| Straight after index | | | | | |
| Patients with 1 *Borrelia burgdorferi infection* | 2,088 | 269 | 12.9 | 12 | 0.6 |
| Patients with 2 *Borrelia burgdorferi* infections | 37 | 12 | 32.4 | 0 | 0.0 |
| Patients with 3 *Borrelia burgdorferi* infections | 4 | 0 | 0.0 | 0 | 0.0 |
| Patients with 4 *Borrelia burgdorferi* infections | 1 | 1 | 100.0 | 0 | 0.0 |
| 6 months after index | | | | | |
| Patients with 1 *Borrelia burgdorferi infection* | 1,945 | 218 | 11.2 | 9 | 0.5 |
| Patients with 2 *Borrelia burgdorferi* infections | 37 | 10 | 27.0 | 0 | 0.0 |
| Patients with 3 *Borrelia burgdorferi* infections | 4 | 0 | 0.0 | 0 | 0.0 |
| Patients with 4 *Borrelia burgdorferi* infections | 1 | 1 | 100.0 | 0 | 0.0 |
| ^a^for the analysis 6 months after index, excludes patients censored before 6 months; ^b^symptoms of fatigue, post-viral fatigue, or chronic fatigue syndrome; | | | | | |
