# Supplementary material for: Incidence of Lyme disease in the United Kingdom and association with fatigue: A population-based, historical cohort study
Source: PLoS One. 2022 Mar 23;17(3):e0265765. doi: 10.1371/journal.pone.0265765 (PMC8942220; doi:10.1371/journal.pone.0265765)
Supplement: Attachment — Submitted filename: Response to reviewers.docx [file pone.0265765.s006.docx]

PLOS One

11th November 2021

To the editor and the reviewers,

RE: point-by-point reply to the comments received

Thank you for reviewing the article entitled “Incidence of Lyme disease in the United Kingdom and association with fatigue: a population-based, historical cohort study”.

You can find below a point-by-point reply to the comments received.

**Review Comments to the Author**

*Reviewer #1: In the current manuscript, authors have conducted a survey on Lyme disease patients, diagnosed during 2000-2018, and tried to find out its possible association with long-term fatigue. The authors have used large number of datasets for analysis, which suggested the strong association between Lyme disease and fatigue or ME/CSF. Overall, the current study provides new insights about Lyme disease and it will be useful for future Lyme disease guideline management. However, there are few minor points which should be addressed-

1.* *Incorporate information regarding Post-Treatment Lyme Disease Syndrome (PTLDS) in discussion section as it is often misdiagnosed with chronic fatigue syndrome.*

Reply: we have added a paragraph in the discussion to bring to the reader’s attention this possible misdiagnosis and have added the following references: Nemeth et al., 2016; Gaudino et al., 1997; Schutzer et al. 2011 (see full references below).

2. *Add more information about Chronic fatigue syndrome as it is very complex condition which can be triggered by combination of factors. Even there is no single test available to confirm chronic fatigue syndrome.*

Reply: We have added a paragraph in the introduction section to give more information on Chronic Fatigue Syndrome supported by the following additional references: Estévez-López et al, 2020; Pheby et al, 2020; Carruthers et al., 2011 ; Institute of Medicine (IOM), 2015 (see full references below).

*3. The quality of figure1and 2 is poor, please improve it. Figure 2 is not even readable.*

Reply: The Preflight Analysis and Conversion Engine (PACE) digital diagnostic tool has been used to generate a new copy of all figures and these high-resolution figures have now been uploaded.

*4. Please italicize the Bacterium name mentioned throughout the manuscript*.

Reply: The text *Borrelia burgdorferi* has been italicized throughout the manuscript as requested

*Reviewer #2: The manuscript entitled “Incidence of Lyme disease in the United Kingdom and association with fatigue: a population-based, historical cohort study” with the number PONE-D-21-25917 has been evaluated. It is a retrospective cohort study. The authors determined the incidence of Lyme disease (LD) and the effect of LD on fatigue symptoms using the accumulated data of a large population in UK, between 2000-2018.
In the manuscript, 2,130 patients with a diagnosis of Lyme disease were investigated in comparison with the control group of non-Lyme disease population constituted of 4 times the quantity of Lyme patients. The targeted Lyme disease patients were selected based on appropriate criteria. The control group population was selected based on the similar features that of Lyme disease patients. The study population is sufficient in terms of the quantity and the features for statistical analysis. These are a few of the positive aspects of the investigation. In addition, the association of Lyme disease with fatigue-related symptoms and diagnose of myalgic encephalomyelitis/chronic fatigue syndrome (ME/CFS) were investigated at least for 3 years after Lyme disease diagnosis. The effect of various variables such as age, sex, season, obesity and antibiotic therapy were evaluated in terms of fatigue cases determined in Lyme disease patients. Antibiotic therapy was found as an important covariable that would cause fatigue in the treated Lyme patients. Depending on the frequency of the doctor visit of Lyme patients, fatigue was increasingly detected in the patients that visited the clinics more often. Therefore, healthcare utilisation frequency was found as one of the major variables. Seasonal changes were also found to be important for fatigue symptoms. Effect of Lyme disease on fatigue was demonstrated as higher during autumn and winter.
In this manuscript, demonstration of the persistence of the fatigue symptoms and also ME/CFS symptoms for more than 6 months (though decreased) following Lyme disease diagnosis are interesting findings. Evaluation of the data of a large number of Lyme disease patients in comparison with higher number of comparators increased reliability.*

Reply: Thank you for your comments – no point seemed to require action from our side.

**Overall Comments**

*1. Please ensure that your manuscript meets PLOS ONE's style requirements, including those for file naming. The PLOS ONE style templates can be found at*

[*https://journals.plos.org/plosone/s/file?id=wjVg/PLOSOne_formatting_sample_main_body.pdf*](https://journals.plos.org/plosone/s/file?id=wjVg/PLOSOne_formatting_sample_main_body.pdf) *and*

[*https://journals.plos.org/plosone/s/file?id=ba62/PLOSOne_formatting_sample_title_authors_affiliations.pdf*](https://journals.plos.org/plosone/s/file?id=ba62/PLOSOne_formatting_sample_title_authors_affiliations.pdf)

Reply: The manuscript has been revised according to the style templates, with main changes including updates of affiliations and addition of corresponding author, additions of level1-2-3 headings, figure captions, and references to supplementary information. Please note that IQVIA, the affiliation from the first and second authors, doesn’t have a full form as such [“I” was initially taken from IMS Health or can be interpreted as Intelligence, “Q” initially comes from Quintiles or can be interpreted as Quotient and VIA is basically the path of transformation]. Since the name of this company can’t be spelt out it remains in capitals in the affiliation section.

2. *In ethics statement in the manuscript and in the online submission form, please provide additional information about the patient records/samples used in your retrospective study. Specifically, please ensure that you have discussed whether all data/samples were fully anonymized before you accessed them and/or whether the IRB or ethics committee waived the requirement for informed consent. If patients provided informed written consent to have data/samples from their medical records used in research, please include this information.*

Reply: The following text has been added to the manuscript: “Patients are informed of the data collection scheme by the practice and have the ability to opt-out of the database at any time.”

This information is also now explained in the online submission form, along with the reference number of the UK Research Ethics Committee’s approval of the data collection scheme.

*3. We note that you have included the phrase “data not shown” in your manuscript. Unfortunately, this does not meet our data sharing requirements. PLOS does not permit references to inaccessible data. We require that authors provide all relevant data within the paper, Supporting Information files, or in an acceptable, public repository. Please add a citation to support this phrase or upload the data that corresponds with these findings to a stable repository (such as Figshare or Dryad) and provide and URLs, DOIs, or accession numbers that may be used to access these data. Or, if the data are not a core part of the research being presented in your study, we ask that you remove the phrase that refers to these data.*

Reply: A new supplementary table has been added (S4 Table. Incidence of any types of fatigue and ME/CFS in patients with multiple infections with bacterium *Borrelia burgdorferi*) to support this statement. Additionally, S3 Table has been added to support an earlier statement (in the first section of the discussion) and provide results related to incidence rates, crude and adjusted hazard ratios of any types of fatigue and ME/CFS for Lyme disease using an extended cohort including also suspected Lyme disease patients and their matched controls.

4. *Please include captions for your Supporting Information files at the end of your manuscript, and update any in-text citations to match accordingly. Please see our Supporting Information guidelines for more information:* [*http://journals.plos.org/plosone/s/supporting-information*](http://journals.plos.org/plosone/s/supporting-information)*.*

Reply: We apologize for not doing so in the first version of the manuscript – captions have now been added and citations updated.

5. *Please upload a new copy of Figure 2 as the detail is not clear. Please follow the link for more information: https://blogs.plos.org/plos/2019/06/looking-good-tips-for-creating-your-plos-figures-graphics/"* [*https://blogs.plos.org/plos/2019/06/looking-good-tips-for-creating-your-plos-figures-graphics/*](https://blogs.plos.org/plos/2019/06/looking-good-tips-for-creating-your-plos-figures-graphics/)

Reply: The Preflight Analysis and Conversion Engine (PACE) digital diagnostic tool has been used to generate a new copy of all figures and these high-resolution figures have now been uploaded.

6. *Please review your reference list to ensure that it is complete and correct. If you have cited papers that have been retracted, please include the rationale for doing so in the manuscript text, or remove these references and replace them with relevant current references. Any changes to the reference list should be mentioned in the rebuttal letter that accompanies your revised manuscript. If you need to cite a retracted article, indicate the article’s retracted status in the References list and also include a citation and full reference for the retraction notice.*

Reply: The list of references has been carefully reviewed and, although one cited paper triggered a response (reference 15), none seems to have been retracted. A few papers have been added in the reference list – most of them to address the reviewers’ comments. The complete list of newly added papers can be found here:

Carruthers BM, van de Sande MI, De Meirleir KL, Klimas NG, Broderick G, Mitchell T, et al. Myalgic encephalomyelitis: International Consensus Criteria. J Intern Med. 2011 Oct;270(4):327–38.

Committee on the Diagnostic Criteria for Myalgic Encephalomyelitis/Chronic Fatigue Syndrome, Board on the Health of Select Populations, Institute of Medicine. Beyond Myalgic Encephalomyelitis/Chronic Fatigue Syndrome: Redefining an Illness [Internet]. Washington (DC): National Academies Press (US); 2015 [cited 2021 Nov 2]. (The National Academies Collection: Reports funded by National Institutes of Health). Available from: http://www.ncbi.nlm.nih.gov/books/NBK274235/

Estévez-López F, Mudie K, Wang-Steverding X, Bakken IJ, Ivanovs A, Castro-Marrero J, et al. Systematic Review of the Epidemiological Burden of Myalgic Encephalomyelitis/Chronic Fatigue Syndrome Across Europe: Current Evidence and EUROMENE Research Recommendations for Epidemiology. J Clin Med. 2020 May 21;9(5):E1557.

Pheby DFH, Araja D, Berkis U, Brenna E, Cullinan J, de Korwin J-D, et al. The Development of a Consistent Europe-Wide Approach to Investigating the Economic Impact of Myalgic Encephalomyelitis (ME/CFS): A Report from the European Network on ME/CFS (EUROMENE). Healthc Basel Switz. 2020 Apr 7;8(2):E88.

Nemeth J, Bernasconi E, Heininger U, Abbas M, Nadal D, Strahm C, et al. Update of the Swiss guidelines on post-treatment Lyme disease syndrome. Swiss Med Wkly. 2016;146:w14353.

Gaudino EA, Coyle PK, Krupp LB. Post-Lyme syndrome and chronic fatigue syndrome. Neuropsychiatric similarities and differences. Arch Neurol. 1997 Nov;54(11):1372–6.

Schutzer SE, Angel TE, Liu T, Schepmoes AA, Clauss TR, Adkins JN, et al. Distinct cerebrospinal fluid proteomes differentiate post-treatment lyme disease from chronic fatigue syndrome. PloS One. 2011 Feb 23;6(2):e17287.

We hope you will now find our manuscript ready for publication and are looking forward to receiving your decision.

Yours Sincerely,

Dr Florence Brellier

IQVIA

210 Pentonville Road

LONDON

N1 9JY

United Kingdom

[florence_brellier@hotmail.com](mailto:florence_brellier@hotmail.com)

+447825722790
